# Supplementary material for: “When the pain is so acute or if I think that I’m going to die”: Health care seeking behaviors and experiences of transgender and gender diverse people in an urban area
Source: PLoS One. 2021 Feb 23;16(2):e0246883. doi: 10.1371/journal.pone.0246883 (PMC7901746; doi:10.1371/journal.pone.0246883)
Supplement: Attachment — Submitted filename: Response to Reviewers.docx [file pone.0246883.s001.docx]

**Response to Reviewers**

**1. Please ensure that your manuscript meets PLOS ONE's style requirements, including those for file naming. The PLOS ONE style templates can be found at**

Thank you. We have revised based on your style guide.

**2. We note you have included a table to which you do not refer in the text of your manuscript. Please ensure that you refer to Table 1 in your text; if accepted, production will need this reference to link the reader to the Table.**

Table 1 is now referred to in the text.

**3. We note that you have indicated that data from this study are available upon request. PLOS only allows data to be available upon request if there are legal or ethical restrictions on sharing data publicly. For information on unacceptable data access restrictions, please see** [**http://journals.plos.org/plosone/s/data-availability#loc-unacceptable-data-access-restrictions**](http://journals.plos.org/plosone/s/data-availability#loc-unacceptable-data-access-restrictions)**.**

We sought guidance from our Office of Human Research (OHR). Because our informed consent indicated that only the study team would have access to transcripts and due to the sensitive nature of the interviews, the OHR indicated that transcripts should not be shared.

**In your revised cover letter, please address the following prompts: a) If there are ethical or legal restrictions on sharing a de-identified data set, please explain them in detail (e.g., data contain potentially identifying or sensitive patient information) and who has imposed them (e.g., an ethics committee). Please also provide contact information for a data access committee, ethics committee, or other institutional body to which data requests may be sent.**

We have provided a revised cover letter indicating the IRB’s concerns.

**We will update your Data Availability statement on your behalf to reflect the information you provide.**

Thank you.

**A summary table is suggested.**

A summary table has been provided (Table 2 on pages 7-8 of the clean manuscript). The prior long table was removed and examples incorporated into the Results section. The deletion of Table 2 does not show up in tracked changes, but the modification is apparent in the additional narrative in the Results section (pages 8-15). Table 3 (misnumbered previously as Table 4, now page 16) was retained to succinctly illustrate the themes unique to TGD experience. We felt this short table was easier for a reader than the long table of results that has now been incorporated into the narrative of the results section.

**More discussion is needed on how this study compares with earlier literature.**

Additional context for how the study compares to prior literature has been added to the discussion (pages 18-22).

**Reviewer #2: Thanks for choosing to work on the marginalized community. I find that using both transmasculine/feminine and transgender man/feminine terms inter-changeably may be confusing for the audience not familiar with the transgender population literature and terminology.**

Thank you for this feedback. The original language was intentional in order to indicate that trans experience occurs over a wide spectrum. However, we have changed the terminology to eliminate confusion with the exception of “Transmasculine” in the name of a community organization, since that is a proper noun.

**It would enrich the manuscript to first do a table with all the themes that emerged, then detail thick description of the themes with the excepts from the participants. Further under discussion, it would be good to contrast the findings of this study, with what is out there in terms of literature that supports or disputes your findings.**

A summary table has been provided (Table 2). Other tables have been removed and examples incorporated into the Results and Discussion sections. Additional context for how the study compares to prior literature has been added to the discussion (pages 20-22).
